# Role of Investment Heterogeneity in the Cooperation on Spatial Public Goods Game

**DOI:** 10.1371/journal.pone.0091012

**Published:** 2014-03-14

**Authors:** Wu-Jie Yuan, Cheng-Yi Xia

**Affiliations:** 1 College of Physics and Electronic Information, Huaibei Normal University, Huaibei, China; 2 Key Laboratory of Computer Vision and System (Ministry of Education) and Tianjin Key Laboratory of Intelligence Computing and Novel Software Technology, Tianjin University of Technology, Tianjin, China; University of Maribor, Slovenia

## Abstract

Public cooperation plays a significant role in the survival and maintenance of biological species, to elucidate its origin thus becomes an interesting question from various disciplines. Through long-term development, the public goods game has proven to be a useful tool, where cooperator making contribution can beat again the free-rides. Differentiating from the traditional homogeneous investment, individual trend of making contribution is more likely affected by the investment level of his neighborhood. Based on this fact, we here investigate the impact of heterogeneous investment on public cooperation, where the investment sum is mapped to the proportion of cooperators determined by parameter 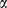. Interestingly, we find, irrespective of interaction networks, that the increment of 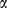 (increment of heterogeneous investment) is beneficial for promoting cooperation and even guarantees the complete cooperation dominance under weak replication factor. While this promotion effect can be attributed to the formation of more robust cooperator clusters and shortening END period. Moreover, we find that this simple mechanism can change the potential interaction network, which results in the change of phase diagrams. We hope that our work may shed light on the understanding of the cooperative behavior in other social dilemmas.

## Introduction

Understanding the sustenance and emergence of cooperation within groups of egoistic agents represents one of long-standing puzzles across various disciplines [1], [2]. A theoretical framework that sheds the light into this open question is the evolutionary game theory [3], [4]. Although the prisoner's dilemma game (PDG) and snowdrift game (SDG), as the metaphors, are often chosen to characterize the social dilemma through the pairwise interactions [5]–[19], the coordination and management of many common resources, such as environment problems, climate change, public traffic and financial markets, needs to be implemented and solved by multiple sides or group interactions, which is best described by the so-called public goods game (PGG) [20]–[29]. In the PGG model [30], any participant simultaneously decides to contribute (i.e., cooperate) or not contribute (i.e., defect) to the public pool. After that, all contributions of cooperators are summed and multiplied by a synergy factor 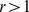, and then uniformly distributed among all participants regardless of their contributions. Nevertheless, in this situation, there is a free-ride problem [31]: why an individual makes contribution if he can reap the benefits without bearing any cost, and why a agent chooses cooperation if he can obtain higher payoff by defecting. Hence, the game theory often predicts a very pessimistic conclusion: all participants take the defective strategy and it eventually leads to the “tragedy of commons” whereby nobody contributes [32].

However, to our surprises, the above-mentioned prediction is different from many experimental findings in the literature [33]. In fact, from the perspectives of the whole group or population, the total cooperation is obviously optimal since it creates the greater collective benefits. Thus, several mechanisms have been put forward to illustrate the evolution of cooperation on the public goods games. For example, a viable mechanism to facilitate the public cooperation is to punish the non-cooperating participants [34]–[37], but it can also evoke the so-called second-order free rider problem in which the investment of publishers may be exploited by cooperators [38]. Meanwhile, volunteering or voluntary participation [39], reputation mechanism [40] and other theoretical supplements [20] are also proposed as effective means to promote the cooperation on spatial public goods games. In addition, the heterogeneous topology has been ubiquitously found in many natural, social, biological and engineering systems, and this structural heterogeneity creates the social diversity within the system which may greatly promote the collective cooperation [41].

Except for the well-mixing assumption, in reality the spatial PGG also draws a lot of attention of statistical physicists who focused on the phase transition behaviors in the evolution of cooperation [20]. The results indicate that the pattern formation or agglomeration may be responsible for the public cooperation on the spatial PGG [42]. Interestingly, the nonhomogeneous teaching activities play an important role in the promotion of cooperation for the spatial public goods games [43]. Importantly, noise taking place in the strategy adoption can noticeably influence the cooperative behaviors and even substantially change the outlay of cooperators' density in dependence on the noise intensity [44]–[50]. Qualitatively similar findings are also discovered in the impact of diversity on the cooperation of PGGs [51]. In particular, Szolnoki et al [52] make use of two different types of regular graph, square and honeycomb lattice, to explore the impact of interaction topology and group size on the cooperation in spatial PGG. It is shown that increasing group size leads to indirect linkage or interaction among players participating in the same PGG group, further changes the effective interaction topology and substantially alters the cooperation behaviors on regular lattice without any overlapping structures. However, in most works, the contribution of a cooperator to all PGG groups is still assumed to be identical, which is far from many realistic situations: heterogeneous activity [20]. Inspired by this fact, an interesting question takes place: if we allow cooperators hold heterogeneous investment, how does cooperation varies?

Aiming to answer this question, in this work we consider a spatial PGG model, where the cooperator's contribution will be non-uniformly distributed over all PGG groups it involves. While its contribution to each group can be mapped to a function of proportion of cooperations tuned by the parameter 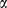. Large-scale numerical simulations demonstrate that the non-homogenous distribution of cooperator's contribution will largely promote the evolution of cooperation. This trait becomes more obvious with the increment of 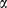, irrespective of interaction networks. Moreover, we also find that this mechanism changes the potential interaction networks.

## Methods

Here, we consider the spatial PGG: all participants are placed on a regular interaction graph which is a square lattice with Von Neumann neighborhood as described in Ref.[52]. Each player will participate in 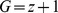 PGG groups where one PGG group is centered around the focal player and other 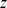 ones are correspondingly centered around 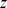 nearest neighbors. Among them, 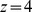 is the size of neighborhood, coordination number or number of all nearest neighbors, and 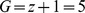.

Initially, each participant or player on site 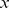 is equally probably designated as a contributor (cooperator, 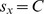) or a non-contributor (defector, 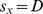). Then, each player will participate in 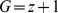 PGG groups and collect its payoff 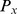 by accumulating the respective share of each PGG group to which it belongs. In each PGG group, without loss of generality, the contribution of a defector is always fixed to be 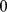 while the contribution of a cooperator is not 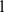, which is the general assumption in many previous reports [34]–[37], [53]. Here, we hypothesize that the contribution or investment of a cooperator into a PGG group in which it participates depends on the fraction of cooperators inside that group, and this investment quantity 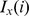 can be depicted as follows,
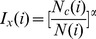
(1)where 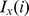 stands for the investment of a cooperator 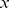 into the 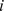 centered PGG group, 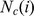 and 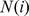 represent the number of cooperators and all players inside 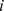-centered PGG group. 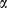 is a tunable parameter which characterizes, to some extent, the heterogeneity of distribution of a cooperator's contribution. Noticeably, 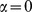 leads to the traditional PGG model [34]–[37], [53], and 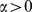 ensures that the investment will be beneficial to the PGG group with higher cooperation level. After that, the investment will be summarized from all cooperators inside 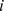-centered PGG group and multiplied by an enhancement factor 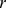. At last, the amplified investment will be evenly allocated among all players inside that group. Thus, the net payoff of player 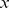 from the 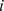-centered PGG group will be equal to
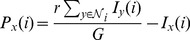
(2)where 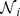 denotes the set of player 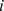 and its 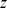 nearest neighbors. Subsequently, the total payoff 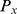 of player 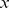 can be calculated as follows,
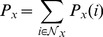
(3)


Since the system evolves according to the Monte Carlo Simulation (MCS) procedure, the player's strategy will be updated by a specific rule after acquiring the total payoff. Here, within one elementary MCS time step, we will randomly choose a player 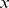 and one of its nearest neighbors 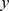, and calculate their total payoffs 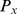 and 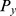 based on the above-described order. Then, player 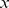 will update its strategy 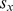 by adopting the strategy 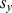 of player 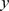 with the following Fermi probability,
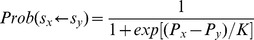
(4)where 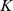 characterizes the uncertainty during the strategy imitation [44], [45]. For 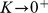, the player 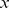 will deterministically take the strategy of player 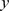 if 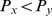. While for 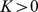, player 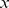 can also imitate the strategy of player 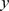 even if player 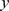 performs worse as far as the total payoff is concerned, which can mimic the error or non-rationality during the course of decision making to a certain extent. In addition, the strategy is asynchronously updated during the system evolution, that is, the strategy of each player can only have a chance to be updated on average within one full MCS time step.

## Results

**Figure 1:**
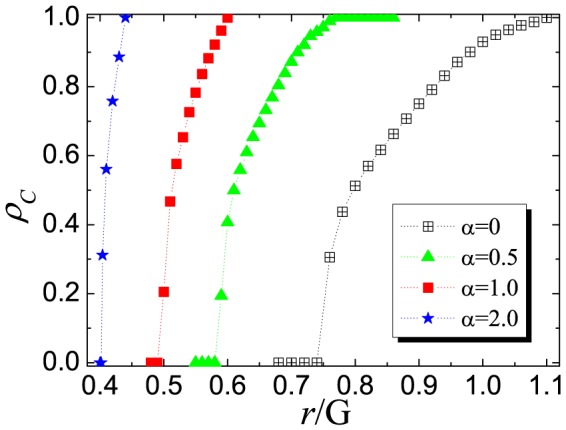
Fraction of cooperators 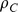 as a function of normalized enhancement factor 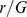 on square lattice with Von Neumann neighborhood. Here, 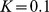, 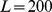 and other linear sizes 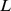 are also verified not to change the qualitative results of our PGG model. Among them, the fraction of cooperators 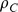 within the whole population is averaged over 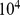 MCS time step after discarding the transient steps up to 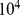 MCS.

We start by presenting how the heterogeneous investment affects the evolution of cooperation on spatial cooperations. Figure 1 depicts the dependence of 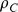 on the normalized enhancement factor 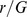 for different tunable parameters 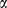. It is obvious, compared to the standard case (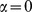), that the evolution of cooperation is greatly promoted when 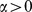. We can observe the emergence of cooperation from two sides: on one hand, the transition point 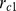 of normalized synergy factor (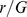) between the full defection (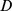) and mixing state of cooperators and defectors (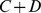) is largely reduced, and it means that the players easily transcend the barrier of defection and are prone to cooperate under the low cooperation cost; On the other hand, the critical point 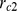 from the mixing state (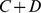) into the full cooperation (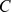) has also become smaller than that in the traditional case. In particular, the larger 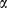, the smaller the critical point. The two critical points are nearly same when 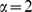 and it means the transition from the full defection into the full cooperation is almost abrupt. In addition, according to Eq.(1), our PGG model with a tunable parameter 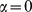 is reduced into the standard case in which each cooperator will make a constant investment quantity, for example, to be 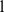 in Ref.[52]. Meanwhile, our simulation results with 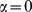 are totally identical with those in [52]. Thus, we can conclude that the investment heterogeneity of a cooperator into different PGG groups are responsible for the promotion of cooperation.

**Figure 2:**
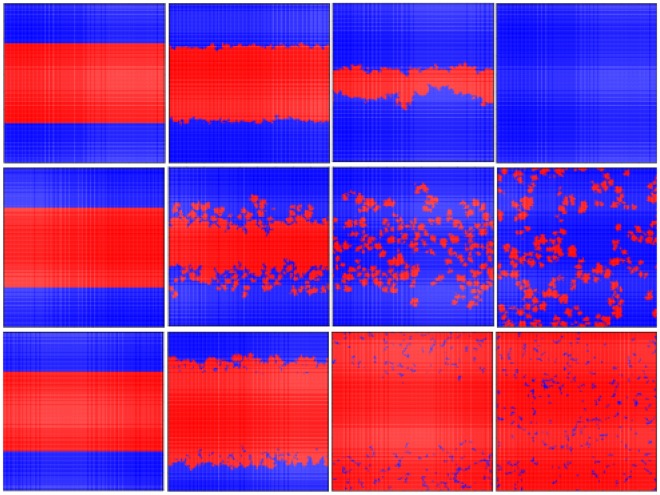
Characteristic snapshots of cooperators (red) and defectors (blue) under the prepared initial state for different times steps. From top row to bottom panel, the tunable parameter 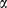 is set to be 0, 0.5 and 1.0, respectively. In all dynamical patterns, the synergy factor 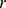 is 0.6, lattice size 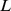 is 200 and strategy adoption uncertainty 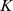 is 0.1.

To further illustrate the origin favoring the cooperation in our PGG model, Figure 2 depicts the evolution of characteristic snapshots for three different tunable parameters 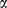 under the prepared initial state. Here each row of patterns correspond to a different 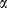, and four columns denote the pattern at 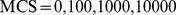, respectively. Under these three cases, the initial distribution of cooperators and defectors are totally same, in which all cooperators are deployed in the middle row of the lattice and defectors are placed on two sides of the lattice. However, the evolutionary pattern exhibits the distinct features for different 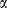 after enough time steps (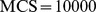). In the standard case (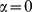), the investment will be shared in all PGG groups and the free-ride from the defectors thrives so that the cooperators cannot resist the explorations of defectors and tend to be extinct in the end. While for 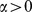, the cooperator's investment will be heterogeneously allocated among 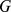 PGG groups, the PGG group with higher cooperation level will obtain the more investment and then this effect will further encourage more players to take the cooperative strategy within this group. The larger 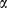, the stronger this effect, hence the cooperators have enough power to create the cooperative (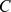) clusters to inhibit the invasion of defectors. Moreover, the fraction of cooperators in the population will become higher and higher as 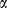 increases.

**Figure 3:**
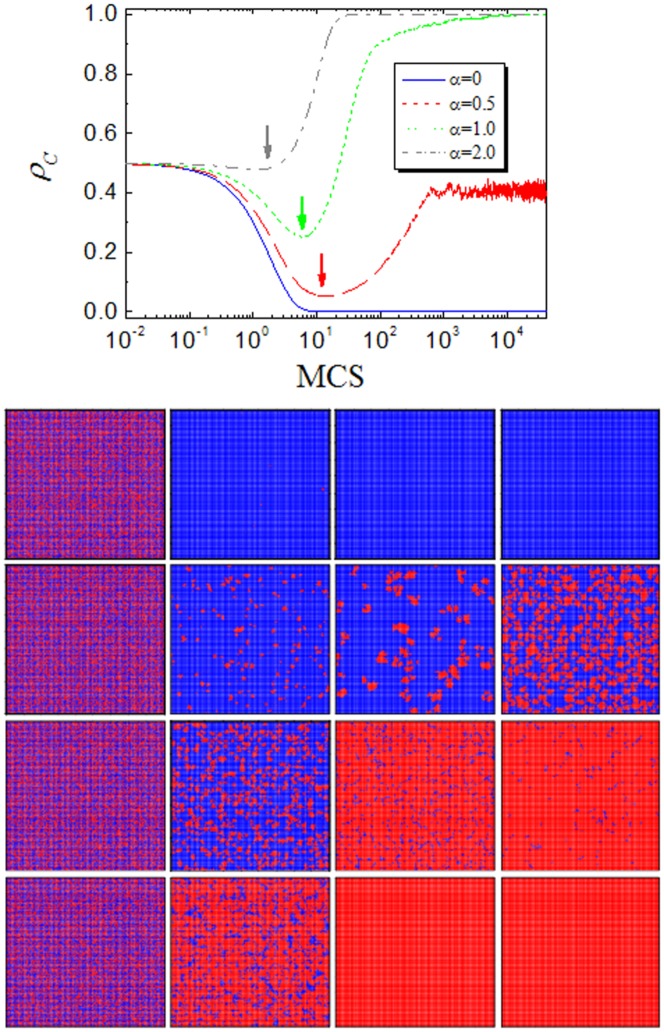
Top panel: time courses depicting the evolution of cooperation for different values of 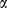 under the random distribution of strategies. Note that the arrows denote the end of enduring (END) period and the beginning of expanding (EXP) period. Bottom panel: the evolution snapshots of different values of 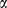. From top to bottom, the values of 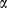 are 0, 0.5, 1.0 and 2.0, respectively. The colore code is the same with Fig.2: cooperator (red) and defector (blue). From left to right, the time steps are 0, 10, 100, 1000 for each value of 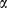.

Moreover, it is also interesting to examine the time course of cooperation under this novel mechanism. According to the recent investigate prediction [19], [54], [55]: a typical evolution process can be divided into two evident periods: the enduring (END) period and the expanding (EXP) period. The shorter the END period (the more perfect the formation of cooperator cluster), the higher the final cooperation level. We expect that under the present mechanism the increment of 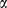 is beneficial for END period and cooperators can converge into more robust clusters. Figure 3 shows the results obtained for 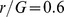 under different 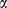 value. What we first glance at is the evolution trend of 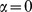 (namely, the traditional version), there exists only END period and cooperators die out. However, with the increment of 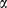, the tide changes: END periods becomes shorter and shorter (the end of END period is marked by arrows), and at the same time the remainder cooperators form clusters and can expand in the subsequent EXP period. Obviously, when 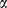 is sufficient large (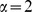), the initial invasion of defection becomes very difficult, hence END periods vanishes and cooperators directly organize clusters. Along this line, the cooperator clusters can expand effectively and reach full cooperation state faster. To further support this point, we also feature the corresponding snapshots under the same time steps. It is obvious that the lager the value of 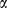, the larger the reminder cooperator cluster at the end of END period, which finally leads to larger clusters. This observation validates our guess: heterogeneous investment can change the evolution trend (i.e., accelerate the END period) and promotes the formation of cooperator clusters, which is the basic guarantee for the maintenance of cooperation.

**Figure 4:**
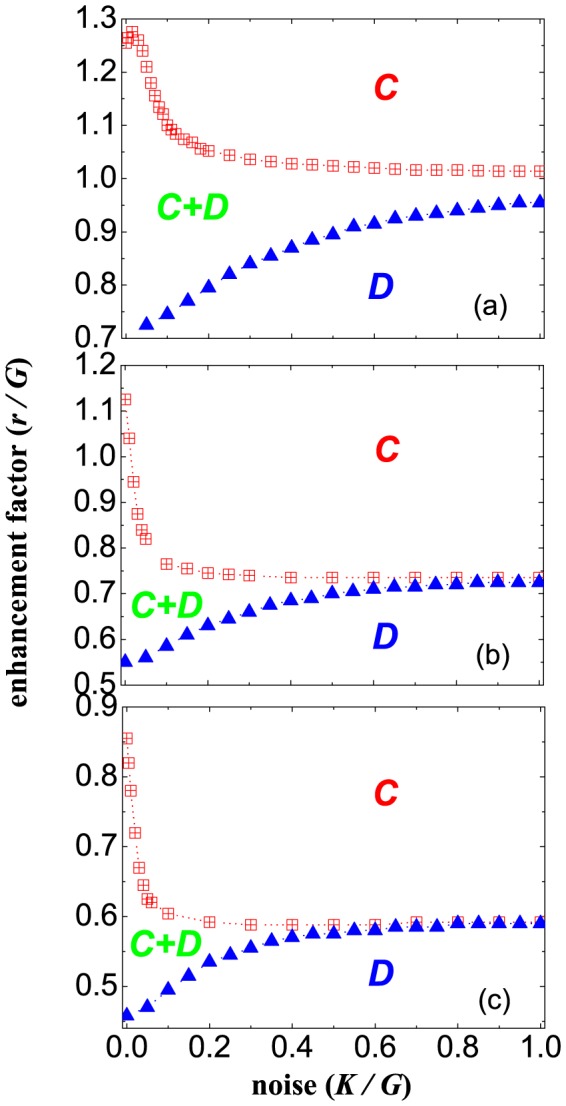
Full normalized 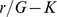 phase diagrams for different tunable parameters 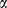. From top to bottom, panels (a), (b), (c) correspond to 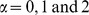, respectively. The lattice size is 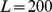 and PGG group size is fixed to be 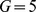.

The robustness of cooperation against the noise can be discussed through the full 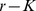 phase diagram in Fig. 4. Here we depict the relationship between critical synergy factor 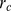 and noise strength 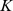 which are both normalized by the group size (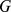). It is observed that the phase boundaries display the distinct behaviors for three different 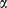. In the traditional case (i.e., 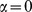), the boundary between the full defection 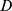 and mixed phase 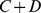 (i.e., lower boundary 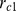) will monotonically decrease as 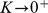, but the boundary separating the mixed phase 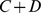 from full cooperation 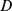 (i.e., upper boundary 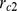) takes on a bell-shaped outlay which means that there is an optimal noise strength ensuring the coexistence of cooperators and defectors. As 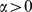, the lower boundary is nearly similar to the traditional one and only the critical value is much smaller, which means the cooperator is easier to sustain in the sea of defectors. However, the upper boundary will continuously increase in the limit of strong selection (i.e. 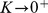), and introducing the noise will benefit the emergence of cooperators. In particular, the upper and lower boundaries will almost collapse into the same curve under the weak selection limit (i.e. 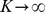), which means that it is impossible for the cooperators and defectors to coexist within the same population. Thus, the heterogeneous investment will lead to the phase transitions which differ from the traditional case, and is responsible for the promotion of cooperation in the spatial PGG model. Moreover, we can observe that optimal cooperation dies out with the increment of 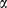, while the actual topology network does not have any change. Similar to the previous literatures [45], [46], we deduce that this may caused by the fact that the topology interaction network has changed. The square lattice obviously lacks overlapping triangles and thus enables the observation of an optimal 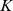, trimming the likelihood of who will act as a investor seems to effectively enhance linkage among essentially disconnected triplets and thus precludes the same observation.

**Figure 5:**
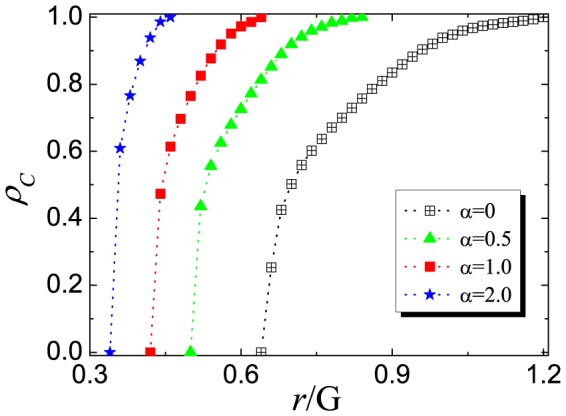
Fraction of cooperators 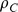 as a function of normalized enhancement factor 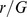 on triangular lattices in which 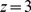 and 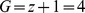 as that in Ref.[52]. Other parameters are identical with those in Fig.1.

Finally, it is still of interest to examining the universality of this simple mechanism under different topologies. To answer this question, in Fig. 5 we consider one type of triangular lattice depicted as in Ref.[52], in which the coordination number 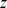 is 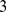 so that each player will participate in 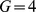 different PGG groups. It can be clearly shown that the promotion of cooperation is very obvious and similar to that on the square lattices. Again, under the heterogeneous payoff distribution mechanism, the fraction of cooperators can be greatly elevated when compared to the traditional PGG model in which the total payoff will be equally allocated within a group. Likely, the critical synergy factors 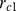 or 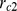 will also be decreased into a lower value, below which the cooperators will completely disappear, or the cooperators and defectors will coexist. Thus, irrespective of the interaction networks, the heterogeneous investment can be regarded as an universally effective in promoting the evolution of cooperation.

## Conclusions and Discussions

To sum, a novel spatial PGG model with heterogeneous payoff investment is proposed to illustrate the public cooperation behavior among selfish agents. Distinguishing from the traditional version, the investment of cooperator for a given PGG group (i.e., 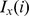) is mapped to the fraction of cooperators inside tuned by a parameter 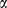. (i.e., 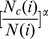). In this case, a cooperator tends to give a larger investment share into a group with the higher cooperation level. On one hand, the cooperative groups will receive the more investment; On the other hand, the cooperator who gives this group will also obtain the higher payoff. This duplicate mechanism to promote the cooperation encourages more players to adopt the cooperation strategy and enhance the collective cooperation. Large scale numerical simulations display that the cooperation can be greatly promoted under the heterogeneous resource investment scheme, regardless of the interaction topology and initial state. Compared with the traditional PGG model, the critical values 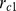 and 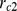 will be largely reduced, which will be beneficial to the cooperators to resist the defective temptation. In particular, the phase diagrams are largely changed and the coexistence region between cooperators and defectors are absent under the weak selection limit. Altogether, current findings help to understand the persistence of cooperation within many real-world systems.
